# Increased expression of vascular endothelin type B and angiotensin type 1 receptors in patients with ischemic heart disease

**DOI:** 10.1186/1471-2261-9-40

**Published:** 2009-08-25

**Authors:** Ivan Dimitrijevic, Marie-Louise Edvinsson, Qingwen Chen, Malin Malmsjö, Per-Ola Kimblad, Lars Edvinsson

**Affiliations:** 1 Department of Medicine, Lund University Hospital, Lund, Sweden; 2 Department of Ophthalmology, Lund University Hospital, Lund, Sweden; 3 Department of Cardiothoracic Surgery, Lund University Hospital, Lund, Sweden

## Abstract

**Background:**

Endothelin-1 and angiotensin II are strong vasoconstrictors. Patients with ischemic heart disease have elevated plasma levels of endothelin-1 and angiotensin II and show increased vascular tone. The aim of the present study was to examine the endothelin and angiotensin II receptor expression in subcutaneous arteries from patients with different degrees of ischemic heart disease.

**Methods:**

Subcutaneous arteries were obtained, by biopsy from the abdomen, from patients undergoing coronary artery bypass graft (CABG) surgery because of ischemic heart disease (n = 15), patients with angina pectoris without established myocardial infarction (n = 15) and matched cardiovascular healthy controls (n = 15). Endothelin type A (ET_A_) and type B (ET_B_), and angiotensin type 1 (AT_1_) and type 2 (AT_2_) receptors expression and function were examined using immunohistochemistry, Western blot and *in vitro *pharmacology.

**Results:**

ET_A _and, to a lesser extent, ET_B _receptor staining was observed in the healthy vascular smooth muscle cells. The level of ET_B _receptor expression was higher in patients undergoing CABG surgery (250% ± 23%; P < 0.05) and in the patients with angina pectoris (199% ± 6%; P < 0.05), than in the healthy controls (100% ± 28%). The data was confirmed by Western blotting. Arteries from CABG patients showed increased vasoconstriction upon administration of the selective ET_B _receptor agonist sarafotoxin S6c, compared to healthy controls (P < 0.05). No such difference was found for the ET_A _receptors. AT_1 _and, to a lesser extent, AT_2 _receptor immunostaining was seen in the vascular smooth muscle cells. The level of AT_1 _receptor expression was higher in both the angina pectoris (128% ± 25%; P < 0.05) and in the CABG patients (203% ± 41%; P < 0.05), as compared to the healthy controls (100% ± 25%). The increased AT_1 _receptor expression was confirmed by Western blotting. Myograph experiment did however not show any change in vasoconstriction to angiotensin II in CABG patients compared to healthy controls (P = n.s).

**Conclusion:**

The results demonstrate, for the first time, upregulation of ET_B _and AT_1 _receptors in vascular smooth muscle cells in ischemic heart disease. These receptors may play a role in the pathophysiology of ischemic heart disease and could provide important targets for pharmaceutical interventions.

## Background

The renin-angiotensin and the endothelin systems are essential in vascular homeostasis and may become maladaptive in cardiovascular diseases [1]. Angiotensin II and endothelin-1 are formed in the endothelium and induce potent vasoconstriction and proliferation of vascular smooth muscle cells [2,3]. The continuous production of endothelin-1 and angiotensin II in the endothelium is important for the control of vessel tone and changes in the endothelin- and renin-angiotensin-systems can give rise to dysfunctional vessels such as those seen in patients with cardiovascular risk factors [4]. Endothelin-1 and angiotensin II have therefore been suggested to play a role in the development if cardiovascular diseases, including hypertension [5], chronic heart failure [6] and atherosclerosis [7].

Endothelin-1 mediates its effects through two distinct G-protein coupled receptors; the endothelin type A (ET_A_) and type B (ET_B_) receptors. During physiologic conditions, the ET_A _receptor is the dominant receptor subtype expressed in vascular smooth muscle cells and mediates contraction, while the ET_B _receptor is primarily located on endothelial cells and mediates vasodilatation via the release of nitric oxide and prostaglandins [8]. ET_B _receptors on vascular smooth muscle cells have however been observed to be upregulated during pathological conditions such as atherosclerosis [9] and congestive heart failure [10]. Endothelin receptors on vascular smooth muscle cells are both mitogenic, leading to atherosclerosis and can induce strong vasoconstriction, resulting in elevated vascular tone that contributes to the development of ischemic cardiovascular disease.

Two angiotensin II receptors have been identified in man, AT_1 _and AT_2 _receptors, which are members of the G-protein coupled seven-transmembrane domain receptor family. The vascular effects of angiotensin II are primarily mediated by AT_1 _receptors located on smooth muscle cells which induce vasoconstriction and mitogenesis. Conversely, AT_2 _receptors are located on endothelial cells and are known to induce vasodilatation, inhibit cell growth and stimulate apoptosis [11]. AT_2 _receptors have been shown, although to a lesser extent, in vascular smooth muscle cells. Angiotensin II acts, apart from being a potent vasoconstrictor also as a growth factor that regulates cell growth, differentiation and fibrosis, as well as being implicated in the pathology of heart failure, hypertension and atherosclerosis [11].

In vivo studies on the effects of endothelin-1 and angiotensin II in the human peripheral vasculature have previously mainly been performed using a forearm blood flow model. To the best of our knowledge, this is the first *in vitro *study using peripheral vascular tissue samples isolated from patients with different degrees of cardiovascular disease. The peripheral vasculature is contributing significantly to total peripheral resistance leading to our use of small peripheral arteries and arterioles, obtained from the subcutaneous tissue in patients. Patients with angina pectoris without established myocardial infarction, patients undergoing coronary artery bypass graft (CABG) surgery because of ischemic heart disease and cardiovascular healthy controls, were included in the study. The expressions of endothelin ET_A _and ET_B_, and angiotensin AT_1 _and AT_2 _receptors in vascular smooth muscle cells were studied by using immunohistochemistry, Western blot and *in vitro *pharmacology.

## Methods

### Ethics

The project was approved by the Ethics Committee of Lund University in Sweden (LU Dnr: 308/2004) and conforms to the principles outlined in the Declaration of Helsinki. Each individual provided written consent to the procedure.

### Study groups

Three different groups of patients were included in the immunohistochemistry part of the study.

1. Cardiovascular healthy controls (controls). This group consisted of ten healthy volunteers without previous history of chest pain, cardiovascular disease or prior cardiac medications. Blood samples for laboratory analysis were taken prior to the biopsy.

2. Fifteen patients with angina pectoris without established myocardial infarction (patients with angina pectoris). These patients were admitted to the medical emergency unit with angina pectoris without signs of prior or ongoing myocardial infarction as measured by electrocardiogram and biomarkers for myocardial injury. The patients had no history of heart failure. Blood samples for laboratory analysis were taken at the telemetry unit after the chest pain had resolved.

3. Patients undergoing coronary artery bypass graft surgery (CABG) (n = 10). These patients underwent elective CABG because of stabile ischemic heart disease confirmed by angiography revealing coronary disease in three arteries. The patients had no prior known myocardial infarction but showed different degrees of heart failure secondary to their heart vessel disorder. Blood samples for laboratory analysis were taken the day before the surgical intervention.

The patient background characteristics are defined in Table 1.

**Table 1:** Physical and Biochemical Characteristics in the Study Subjects

**Variable**	**Control** **n = 10**	**Angina** **n = 15**	**CABG** **n = 10**
Age (years)	62 ± 13	64 ± 13	70 ± 5
Smokers	2	4	1
SBP (mmHg)	125 ± 9	142 ± 15*	140 ± 14*
DBP (mmHg)	72 ± 8	76 ± 9	78 ± 12
Total cholesterol (mg/dl)	4.9 ± 1.6	5.3 ± 1.2	4.4 ± 2.4
LDL (mg/dl)	3.5 ± 0.7	3.5 ± 1.1	2.5 ± 1.6
HDL (mg/dl)	1.5 ± 0.5	1.5 ± 0.4	1.3 ± 0.5
Triglycerides (mg/dl)	1.5 ± 0.9	1.7 ± 1.0	1.9 ± 0.9
Apo lipoprotein A (mg/dl)	1.5 ± 0.3	1.6 ± 0.2	1.3 ± 0.5
Apo lipoprotein B (mg/dl)	0.9 ± 0.2	1.0 ± 0.3	0.9 ± 0.3
NYHA class			
I	10	10	4
II	0	0	4
III	0	0	2
IV	0	0	0
NT pro-BNP (ng/L)	106 ± 112	218 ± 243	1626 ± 2157*
C reactive protein (mg/dl)	2.1 ± 1.9	6.2 ± 15.3*	6.9 ± 6.1*
Beta blockers (n)	0	6	7
Statins (n)	0	5	8
ARB or ACEI (n)	0	3	6
HbA1c (%)	4.5 ± 0.5	4.7 ± 0.4	4.7 ± 0.8
BMI (kg/m^2^)	28 ± 2	29 ± 5	27 ± 3

Values are expressed as mean ± SD. Statistical analysis was performed using ANOVA with Dunnet's post-test for multiple comparisons. Significance was defined as P < 0.05 (*). Abbreviations are: CABG, coronary artery bypass graft surgery; SBP, systolic blood pressure; DBP, diastolic blood pressure; LDL, low-density lipoprotein cholesterol; HDL, high-density lipoprotein cholesterol; NYHA, New York Heart Association Functional Classification; NT-proBNP, N-terminal pro B-type natriuretic peptide; ARB, angiotensin receptor blockers; ACEI, angiotensin converting enzyme inhibitors and BMI, body mass index.

### Tissue collection

A biopsy was taken from the subcutaneous tissue of the abdomen. For the control group and the patient with angina pectoris, this was done during local anaesthesia (2 ml, 1% Xylocain; AstraZeneca, Sweden). For the patients undergoing CABG surgery the biopsy was taken under general anaesthesia during the initiation of surgery. A subcutaneous tissue (1 cm^3^), containing small arteries, was removed from the abdomen. The tissue was frozen in ice-cold isopentane and stored at -80°C.

### Immunohistochemistry

The biopsies were stored at -80°C prior to sectioning into 8-*μ*m-thick slices in a calibrated Microm HM500M cryostat (Microm). The sections were fixed and rehydrated in phosphate-buffered saline (PBS). The sections were incubated overnight with rabbit anti-human ET_B _(16207, IBL) diluted 1:400, goat anti-human ET_A _(sc-21194, Santa Cruz Biotechnologies) diluted 1:50, rabbit anti-human AT_1 _(sc-1173, Santa Cruz Biotechnologies) diluted 1:50, rabbit anti-human AT_2 _(sc-9040, Santa Cruz Biotechnologies) diluted 1:50, mouse anti-human CD-31 (M0823, DAKO) diluted 1:100 and mouse anti-human alpha-actin (M0851, DAKO) diluted 1:1000. All dilutions were done in PBS with 10% fetal calf serum. Antibodies to alpha-actin were used to localize smooth muscle cells and antibodies to CD-31 were used to localize endothelial cells. The secondary antibodies used were donkey anti-rabbit Cy™3 conjugated (711-165-152, Jackson ImmunoResearch) 1:100, donkey anti-goat Cy™3 conjugated (705-165-003, Jackson ImmunoResearch) 1:100 and donkey anti-mouse Texas Red conjugated (715-076-150, Jackson ImmunoResearch) 1:200 in PBS. Only secondary antibodies were used as control. The antibodies were directed against a part of the respective receptor protein; this amino acid sequence was used as a control to block the antigenic site. The data from both immunocytochemistry and Western blot are given at the respective company's home page.

### *In vitro *pharmacology

The resistance arteries were removed from the abdomen from cardiovascular healthy patients undergoing abdominal reduction surgery (n = 5) or during CABG surgery (n = 5). The subjects had the same clinical characteristics as those for immunohistochemistry (Table 1). Vessels were placed in saline buffer on ice, transported to the laboratory and subsequently analyzed for their contractile properties in a myograph recording their isometric tension. The vessels were cut into 1-mm-long cylindrical segments and mounted on two L-shaped metal prongs, unilaterally a force displacement transducer continuously recorded the isometric tension [12]. Mounted vessel segments were immersed in vessel baths at 37°C containing a bicarbonate based buffer solution; NaCl (119 mM), NaHCO_3 _(15 mM), KCl (4.6 mM), CaCl_2 _(1.5 mM), MgCl (1.2 mM), NaH_2_PO_4 _(1.2 mM), and glucose (5.5 mM). The buffer was continuously aerated with oxygen enriched with 5% CO_2 _resulting in pH 7.4. Initially vessels were stretched to a resting tone of 2 mN and was then allowed to stabilize at this tension for 1 h. Contractile capacity of each arterial vessel segment was controlled by exposure to a potassium rich (63.5 mM) buffer solution. Concentration-response curves were obtained by cumulative application of endothelin ET_B _receptor agonist, sarafotoxin 6c at increasing concentrations (10^-11^-10^-6 ^mM), ET-1 (AnaSpec, San Jose, CA) in the concentration range 10^-14 ^to 10^-7 ^M and angiotensin II in the concentration range 10^-12 ^to 10^-6 ^M. Before the application of angiotensin II, the arteries were pretreated with the AT_2 _receptor antagonist PD-123319 (10^-5.5 ^M) for 30 min. After washout, the vessels returned to baseline and endothelin-1 was then added at increasing concentrations (10^-11^-10^-6 ^mM) when endothelin ET_B_receptors were desensitized [13] facilitating endothelin-1 to act solely on the endothelin ET_A _receptors. For details see Nilsson *et al*. [14].

The drugs used, endothelin-1 (ET-1), sarafotoxin 6c (S6c), angiotensin II and PD-123319 were purchased from Sigma Chemical Co (St. Louis, MO). They were dissolved in 0.9% NaCl with 10% albumin.

### Western Blot Analysis

Human vessels (4 from healthy donators and 5 from CABG patients) were frozen in liquid nitrogen and homogenized in cell extract denaturing buffer (BioSource, USA) with addition of a protease inhibitor cocktail (Sigma, USA). Whole cell lysates were sonicated for 2 min on ice, centrifuged at 15,000 × g at 4°C for 30 min, and the supernatants were collected as protein samples. The protein concentrations were determined using the protein assay reagents (Bio-Rad, Hercules, CA, USA) and stored at -80°C until immunoblotting assay. The protein homogenates were diluted 7:2:1(v/v) with 5× LDS Sample Buffer and 10× DTT Reducer (PAGEgel, Inc. San Diego, CA, USA). A total of 40-50 μg of protein was boiled for 5 min and separated by 4-12% SDS Ready Gels (PAGEgel, Inc., USA) for 120 min at 100 v, and transferred electrophoretically to nitrocellulose membranes (Bio-Rad) at 100 v for 60 min. The membrane was then blocked for 1 h at room temperature with phosphate buffered saline (PBS) containing 0.1% Tween-20 (Sigma) and 5% non-fat dried milk, and incubated with sheep anti-ET_B _(Enzo Life Sciences Inc., Farmingdale, NY, USA), rabbit anti-AT_1 _(Abcam plc., Cambridge, UK), or rabbit anti-β-actin (Cell Signaling Technology, Inc., Boston, MA, USA) polyclonal primary antibodies diluted 1:200-500 overnight at 4°C, followed by incubation with anti-sheep (Abcam plc., UK) or anti-rabbit (Amersham Biosciences, Piscataway, NJ, USA) IgG, horseradish peroxidase (HRP)-conjugated secondary antibodies diluted 1: 5000 for 1 h at room temperature. The probed proteins were developed by LumiSensor Chemiluminescent HRP Substrate kit (GenScript Corp., Piscataway, NJ, USA). To detect multiple signals using a single membrane, the membrane was incubated for 5-15 min at room temperature with restored plus Western blot stripping buffer (Pierce Biotechnology, Inc., Rockford, IL. USA). The membranes were visualized using a Fujifilm LAS-1000 Luminiscent Image Analyzer (Stamford, CT, USA.). The quantification of band intensity was analyzed with Image Gauge Ver. 4.0 (Fuji Photo Film Co., LTD., Japan). The expression of target proteins were presented as a relative extent to the level of β-actin and normalized to the percentage of control.

### Calculations and statistics

#### Immunohistochemistry

The experiments were performed using arteries from 15 patients with angina pectoris, 10 patients undergoing CABG surgery and 10 healthy controls. The samples were examined in a fluorescence microscope (Olympus optical Co, LTD, Bx60F5) and the absolute fluorescence intensity was measured with ImageJ . Mean fluorescence values in the area selected were obtained in three sections and at four different regions, and the mean values were calculated. Statistical analysis was performed using ANOVA with Bonferroni's or Dunnet's post-test for multiple comparisons. Significance was defined as P < 0.05 (*). The data are given in the text as percentage difference relative to the mean fluorescence seen in the control. The results are presented as mean values ± standard deviation (SD) and N equals number of patients. Values are presented as percent fluorescence in the angina and CABG groups compared with the control patients group, where the control group is set to 100%.

#### *In vitro *pharmacology

The contractile reaction in each segment is expressed as percentage of the 63.5 mM potassium induced contraction. E_max _value represents the maximum contractile response elicited by an agonist, and the pEC_50_, the negative logarithm of the drug concentration that elicited half the maximum response. Linear regression analysis was applied using values obtained from above and below half-maximum response for calculating the negative logarithm of the concentrations that produced 50% contraction (pEC_50_). Data are presented as mean ± standard error of the mean (S.E.M). Statistical analyses was performed using Student's *t*-test.

#### Western Blot

Comparisons between two groups were performed using two-tailed unpaired Student's *t*-test. A p-value less than 0.05 were considered to be significant. Results were presented as mean ± S.E.M.

## Results

### Endothelin receptor expression

**Figure 1:**
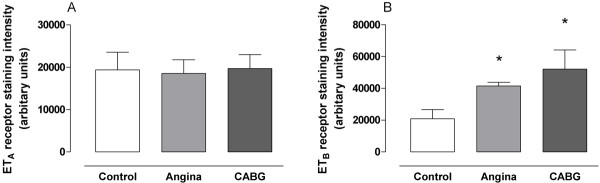
**ET_A _(A) and ET_B _(B) receptor protein expression assessed by immunohistochemistry in human subcutaneous arteries**. Vessles were obtained from patients undergoing coronary artery bypass graft (CABG) surgery because of ischemic heart disease (n = 10), patients with angina pectoris without established myocardial infarction (n = 15) and cardiovascular healthy controls (n = 10). Values are expressed as mean ± SD. Statistical analysis was performed using ANOVA with Dunnet's post-test for multiple comparisons. Significance was defined as P < 0.05 (*).

**Figure 2:**
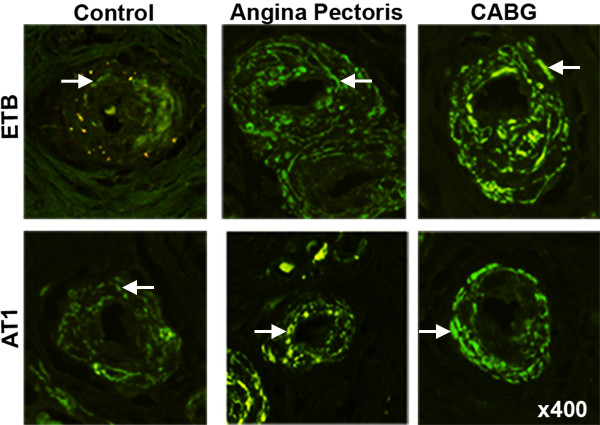
**Representative examples showing immunofluorescence staining experiments for ET_B _and AT_1 _receptors in human subcutaneous arteries**. Vessels were obtained from patients undergoing coronary artery bypass graft (CABG) surgery because of ischemic heart disease (n = 10), patients with angina pectoris without established myocardial infarction (n = 15) and cardiovascular healthy controls (n = 10). Note that the immunostaining intensity for both ET_B _and AT_1 _receptors, indicated with arrows, is higher in the arteries from patients with ischemic heart disease then from healthy controls. Magnification × 400 for all photomicrographs.

In healthy controls, the vascular smooth muscle cells stained for ET_A _and, to a lesser extent, ET_B _receptors. The immunostaining intensities for ET_B _receptors were higher in arteries from the patients undergoing CABG surgery (250% ± 23%; P < 0.05) and from the patients with angina pectoris (199% ± 6%; P < 0.05), than from the healthy controls (Figure 1 and 2). Furthermore, the levels of ET_B _receptor expression were higher in the smooth muscle cell layer in the arteries from the patients undergoing CABG surgery than in the arteries from the patient with angina pectoris (Figure 1B). The levels of ET_A _receptor expression, in the smooth muscle cells, were similar in all three groups (P = n.s.). In endothelial cells ET_B _receptor immunostaining was primarily observed, however, it was not different among the groups.

**Figure 3:**
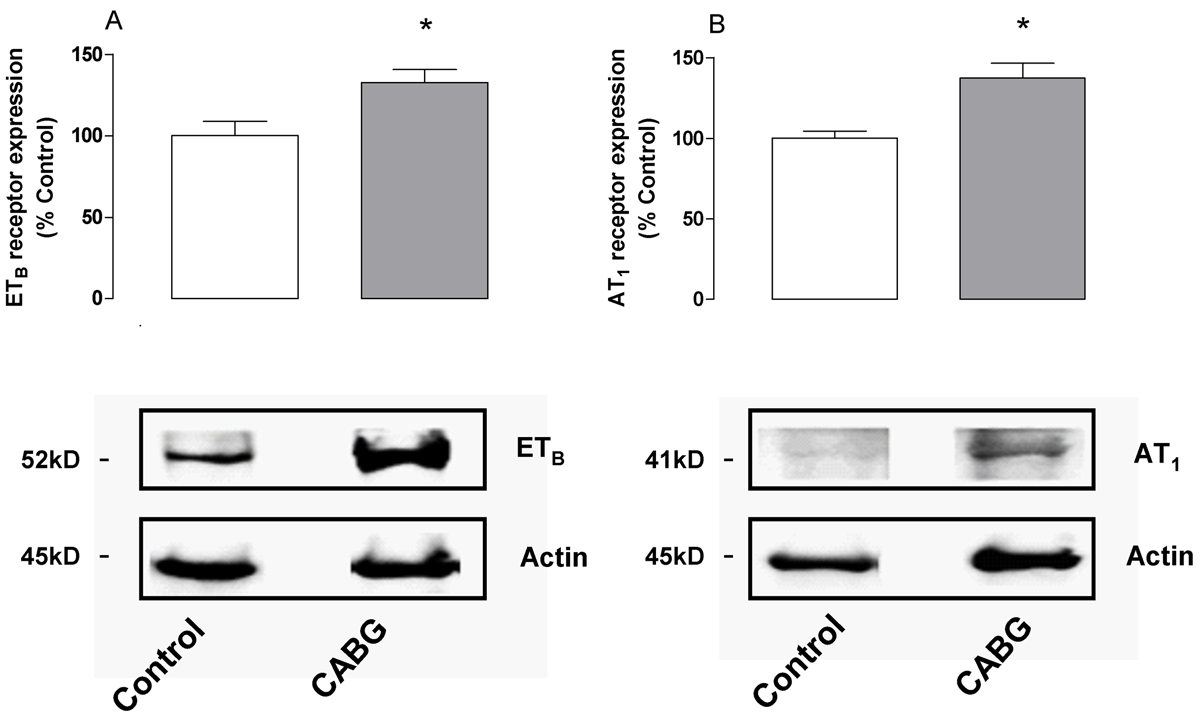
**ET_B _(A) and AT_1 _(B) receptor protein expression in human subcutaneous arteries, examined using Western blot**. Arteries from healthy control patients (n = 4) were compared with arteries from patients undergoing CABG surgery (n = 5). The results are shown as mean values ± S.E.M above the actual Western blot data with beta-actin as control. Statistical analyses was performed using Student's *t*-test, where P < 0.05 (*) was considered significant.

Western blot for ET_B _receptors detected a single band with the approximate molecular weight of 53 kDa in the artery homogenates from all samples including healthy controls and patients undergoing CABG surgery (Figure 3). Densitometric analysis showed that patients undergoing CABG surgery had a mean increase of ET_B _receptors expression (133 ± 8%; P < 0.05), which was significantly more than in healthy controls (Figure 3)

### Angiotensin II receptor expression

**Figure 4:**
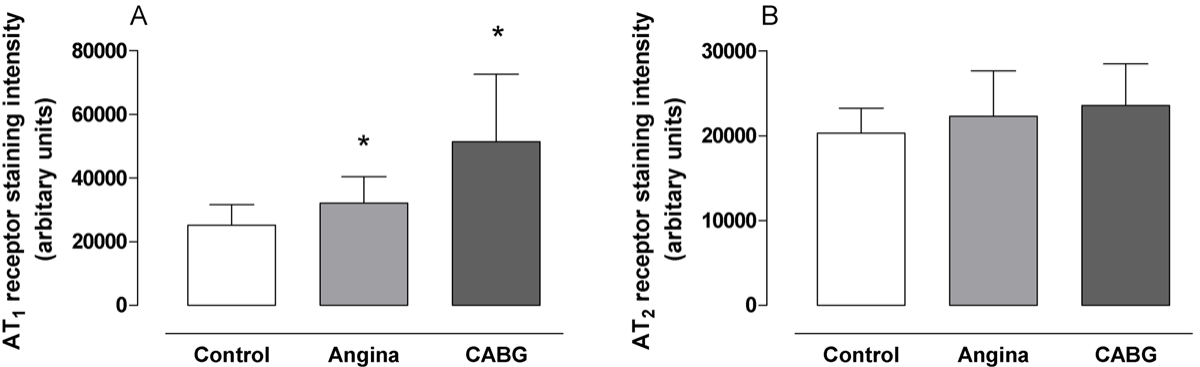
**AT_1 _(A) and AT_2 _(B) receptor protein expression assessed by immunohistochemistry in human subcutaneous arteries**. Vessels were obtained from patients undergoing coronary artery bypass graft (CABG) surgery because of ischemic heart disease (n = 10), patients with angina pectoris without established myocardial infarction (n = 15) and cardiovascular healthy controls (n = 10). Values are expressed as mean ± SD. Statistical analysis was performed using ANOVA with Dunnet's post-test for multiple comparisons. Significance was defined as P < 0.05 (*).

The vascular smooth muscle cells stained for AT_1 _and, to a lesser extent, for AT_2 _receptors. The levels of AT_1 _receptor expression were higher in both the angina pectoris (128% ± 25%; P < 0.05) and in the CABG patients (203% ± 41%; P < 0.05), than from the healthy controls (Figure 2 and 4). In addition, the levels of AT_1 _receptor expression were higher in the smooth muscle cell layer in arteries from patients undergoing CABG surgery than in arteries from patient with angina pectoris (P < 0.05). AT_2 _receptor expression was similar in all three groups. In endothelial cells, only AT_2 _receptor immunostaining was observed. Since the numbers of cells in the endothelial cell layer are limited, no comparisons were made between the groups. Western blot for AT_1 _receptors detected a single band with the approximate molecular weight of 41 kDa in the artery homogenates from all samples including healthy controls and patients undergoing CABG surgery (Figure 3). Densitometric analysis showed that patients undergoing CABG surgery had a mean increase of AT_1 _receptors expression (137 ± 9%; P < 0.05) which was significantly more than in healthy controls (Figure 3).

### Actin and CD-31 immunostaining

**Figure 5:**
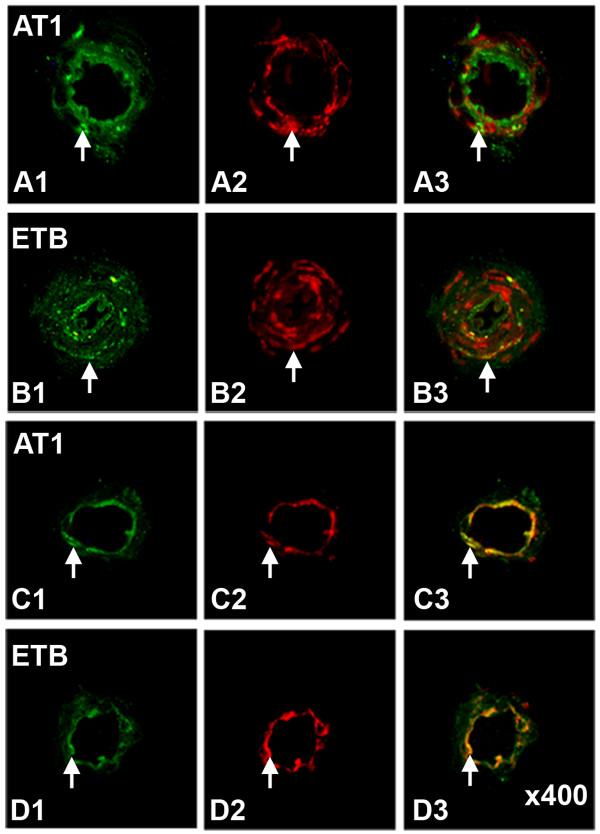
**Co localization of the AT_1 _and ET_B _receptors to vascular smooth muscle cells and endothelial cells**. Immunostaining and co localization of the AT_1 _and ET_B _receptors to alpha-SMC and endothelin in resistance arteries of control patients. Antibodies to AT_1 _(*green*, **A1**, *arrow*) and smooth muscle actin (*red*, **A2 ***arrow*), **A3 **(merged **A1 **and **A2**). Antibodies to ET_B _(*green*, **B1**, *arrow*) and smooth muscle actin (*red*, **B2**, *arrow*), **B3 (**merged B1 and B2). Antibodies to AT_1 _(*green*, **C1**, *arrow*) and CD-31 (*red*, **C2**,*arrow*), **C3 **(merged **C1**+**C2**). Antibodies to ET_B _(*green*, **D1**, *arrow*) and CD-31 (*red*, **D2**, *arrow*), **D3 (**merged **D1 **and **D2**). Magnification × 400 for all photomicrographs.

Double immunostaining showed co localization between on one hand the AT_1 _and ET_B _receptors and on the other hand smooth muscle cell alpha-actin in resistance arteries. As shown in the illustration (Figure 5) there was clear co localization between the two receptor subtypes and alpha-actin both in control and in CABG subjects. CD-31 was used to stain endothelial cells; we observed weak co localization between both receptor and CD31 (Figure 5).

### *In vitro *Pharmacology

**Figure 6:**
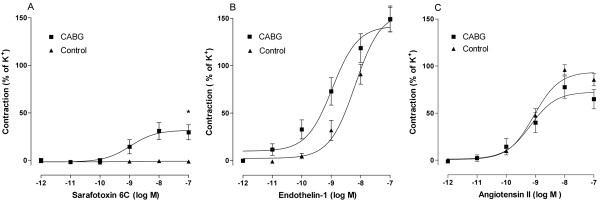
***In vitro *pharmacology**. Concentration-response curves to increasing concentrations of (A) sarafotoxin 6c, (B) endothelin-1, and (C) angiotensin II in arteries from healthy controls (n = 5) and patients undergoing CABG surgery (n = 5). The results are shown as mean values ± S.E.M of five experiments in each group. Statistical analyses was performed using Student's *t*-test, where P < 0.05 (*) was considered significant.

The selective ET_B _receptor agonist S6c was used to study ET_B _mediated contraction. Afterwards ET-1 induced vasoconstriction was studied when endothelin B receptors were desensitized by the previous application of S6c leaving only ET_A _receptors available for a contractile response. Sarafotoxin 6c induced no vasoconstriction in healthy control vessels. On the other hand patients undergoing CABG showed a fairly strong and significant contractile response to the cumulative application of S6c (P < 0.05 n = 5) suggesting upregulation of ET_B _receptors at the functional level (Figure 6A). Endothelin-1 induced a potent vasoconstriction in both healthy controls and in patients undergoing CABG surgery (P = ns) indicating that there were no obvious changes in the ET_A _receptor responses between the two groups (Figure 6B). Angiotensin II induced concentration-dependent contractions of the arteries were strong but there were no differences between the groups (Figure 6C). The potassium rich buffer *(124 *mM) resulted in strong persistent contractions of the resistance arteries with no differences between the groups (data not shown).

### Demographics

The patients' background characteristics are described in Table 1. Taken together, the systolic blood pressure and C reactive protein were higher in the patient with ischemic heart disease (both CABG and angina pectoris). The plasma levels of N-terminal pro B-type natriuretic peptide (NT-proBNP) were higher in the CABG and the angina pectoris groups than in the cardiovascular healthy controls.

## Discussion

### Endothelin receptor expression and function

Our results clearly show that the level of ET_B _receptor expression is higher in the vascular smooth muscle cell layer of arteries from patients with angina pectoris than from healthy controls. The levels of ET_B _receptor expression are even higher in arteries from patients undergoing CABG surgery. We hypothesize that there is a progressive upregulation of this receptor subtype that may correlate with the degree of cardiovascular disease.

Upregulated ET_B _receptors on vascular smooth muscle cells have previously been shown in diabetes, hypertension and in human atherosclerotic coronary arteries and in atherosclerotic plaques [9,15,16]. Plasma levels of ET-1 are elevated in patients with ischemic heart disease and in heart failure; it has been suggested as a prognostic marker [17]. The circulating ET-1 levels are further increased in patients undergoing CABG surgery [18]. This increased activity in the endothelin system may contribute to smooth muscle cell proliferation, vasoconstriction and decreased perfusion in atherosclerotic disease [19-21]. Furthermore, CABG patients had significantly increased pro-BNP levels indicating some degree of heart failure, a condition associated with increased ET_B _receptor mediated systemic vasoconstriction [22]. The elevated endothelin ET_B _contraction in the present study, also verified by Western blot, indicates that the increased levels of contractile endothelin ET_B _receptors on the vascular smooth muscle cells could play an important role in ischemic heart disease since ET_B _receptor activation in healthy controls did not induce any contractions at all.

### Mechanisms underlying the upregulation of ET_B _receptors

The mechanism underlying the increased ET_B _receptor expression is not known, but could depend on increased transcription of ET_B _receptor mRNA triggered by some of the many humeral factors that are increased in ischemic heart disease. Upregulation of ET_B _receptors is known to rely on increased transcription and subsequent translation of receptor mRNA [23]. In the human genome, the 5'-flanking region of the genes encoding the endothelin receptors contain several regulatory elements, like GATA-motifs and E-boxes [24,25]. This indicates that the genes might be activated by for example inflammatory components. Indeed, the presence of interleukin-1β and TNF-α may enhance the upregulation of vascular ET_B _receptors [26]. In agreement, the patients with ischemic heart disease had higher plasma levels of C reactive protein. Atherosclerosis is known to induce a proinflammatory response [27] and this may in part regulate endothelin receptor expression.

### Angiotensin II receptor expression

In the present study, the levels of AT_1 _receptor expression were higher in subcutaneous arteries from patients with ischemic heart disease than in the healthy controls as verified by immunohistochemistry and Western blot. The levels of expression were even higher in arteries from patients undergoing CABG surgery. The plasma level of angiotensin II is known to be elevated in different heart conditions, such as heart failure [28], hypertension [29], hypoxia [30], hypercholesterolemia [31] and hyperglycemia [32]. We have found, herein, that this may be associated with enhanced expression of vascular angiotensin II receptors. Angiotensin II is a potent vasoconstrictor and mitogen of coronary artery smooth muscle cells. The stimulation of AT_1 _receptors results in progression of atherosclerotic lesions, inflammation and plaque rupture. Increased expression of AT_1 _receptors might make the vasculature prone to develop spasm and atherosclerotic plaques, and thus further increase peripheral vascular resistance and reduce lumen. However, we did not observe any enhanced contraction of angiotensin II mediated via AT_1 _receptors as a result of an increased expression in the myograph experiments. One explanation to this could be the increased use of AT_1 _receptor antagonists in patients undergoing CABG, thus blocking the AT_1 _receptors. It is important, however, not to forget that AT_1 _receptors apart from their contractile properties also possess other abilities such as regulating cell growth, differentiation and fibrosis which are important in the pathology of heart failure, hypertension and atherosclerosis.

### Mechanisms underlying the upregulation of AT_1 _receptors

The mechanisms underlying the upregulation of AT_1 _receptor expression in patients with ischemic heart disease cannot be deduced from the present study. Inflammatory mediators, such as IL-1 and IL-6, may upregulate AT_1 _receptors and enhance angiotensin II stimulated vessel hypertrophy [33]. In the present study, the observed plasma levels of C reactive protein was elevated in patients with ischemic heart disease. It has previously been shown that high levels of C reactive protein correlate with high levels of AT_1 _receptor expression [34]. Increased AT_1 _receptor expression has therefore been associated with vascular inflammation. C reactive protein is known to independently predict risk for myocardial infarction, stroke, peripheral artery disease and sudden cardiac death even among apparently healthy individuals [35].

### Clinical perspective

Both ET-1 and angiotensin II have strong impact on cardiovascular diseases as discussed above; herein we will discuss their involvements in other situations: CABG surgery is hampered by deleterious vasospasm in arterial grafts. It has been suggested that impaired vasomotor function may contribute to this vasospasm [36]. Plasma levels of ET-1 and angiotensin II are elevated during CABG surgery [18]. Both endothelin and angiotensin II receptors have been shown to mediate strong vasoconstrictor effects in bypass grafts [28]. Many measures have been undertaken to control the vessel tone during surgery, including dilating the vessels with potent vasodilators such as papaverine, sodium nitroprusside and nifedipine, or mechanically distending the vessels with saline. It might be important to consider endothelin and angiotensin II receptor antagonists for this indication especially since the present results show increased levels of endothelin and angiotensin II receptor expression in patients undergoing CABG surgery.

Angiotensin converting enzyme inhibitors (ACEI) and angiotensin AT_1 _receptor antagonists are frequently being used to prevent the development of target organ damage in atherosclerotic disease and in hypertension [37]. Whether endothelin receptor antagonists will become part of the therapeutic armamentarium in ischemic heart disease remains unclear, and none of these agents is currently being developed for this indication. However, the mixed endothelin blocker Bosentan is used in treatment of pulmonary hypertension [38] and other non-selective ET receptor antagonists have been shown to inhibit the development of atherosclerosis in experimental models [39]. New endothelin antagonists devoid of side effects and perhaps specific for either of the receptor subtypes are discussed in treatment of vasospasm after subarachnoid haemorrhage [40], or alternative inhibitors of the endothelin converting enzymes that generate endothelin-1 may in the future become available to block the endothelin system [5].

### Limitations

The present study has demonstrated increased expression of ET_B _and AT_1 _receptors in patients with ischemic heart disease. However, the patients have confounding factors. The systolic blood pressure was higher in patients with ischemic heart disease compared to the healthy controls. Furthermore, the prevalence of medication with statins, beta blockers, ACEI, and AT_1 _receptor antagonists was common in patients with ischemic heart disease while they were non-existing in our healthy controls. The effect of these confounding factors on the endothelin and angiotensin II receptor expression cannot be concluded from the present study.

## Conclusion

The present study clearly shows that patients with ischemic heart disease have upregulated ET_B _and AT_1 _receptors in the smooth muscle cells of peripheral resistance arteries. The level of ET_B _and AT_1 _expression correlates with the degree of ischemic heart disease, being highest in the patients undergoing CABG surgery. There was no difference in the expression of ET_A _or AT_2 _receptors between the study groups. The changes in ET_B _and AT_1 _receptor expression may be signs of remodeling of the vasculature, which is characteristic of ongoing ischemic heart disease.

## Competing interests

The authors declare that they have no competing interests.

## Authors' contributions

ID planned and executed the immunofluorescence experiments, analyzed the data, preformed the surgical procedure and wrote the manuscript. MLE acquired the patient data, the biopsies and reviewed the manuscript. QC preformed the Western blot experiments and wrote the associated texts. MM participated in the writing of the manuscript. POK performed the thoracic surgical procedure, removed the vessels and reviewed the manuscript. LE conceived and planned the study, and carefully reviewed the data and assisted in writing of the manuscript.

## Pre-publication history

The pre-publication history for this paper can be accessed here:


